# Data supporting the absence of FNR dynamic photosynthetic membrane recruitment in *trol* mutants

**DOI:** 10.1016/j.dib.2016.02.044

**Published:** 2016-02-26

**Authors:** Lea Vojta, Hrvoje Fulgosi

**Affiliations:** Laboratory for Molecular Plant Biology and Biotechnology, Division of Molecular Biology, Institute Ruđer Bošković, 10 000 Zagreb, Croatia

**Keywords:** Photosystem I, FNR, TROL, Dynamic interaction

## Abstract

In photosynthesis, the flavoenzyme ferredoxin:NADP^+^ oxidoreductase (FNR) catalyses the final electron transfer from ferredoxin to NADP^+^, which is considered as the main pathway of high-energy electron partitioning in chloroplasts (DOI: 10.1111/j.1365-313X.2009.03999.x[1], DOI: 10.1038/srep10085[2]). Different detergents and pH treatments of photosynthetic membranes isolated from the *Arabidopsis* wild-type (WT) and the loss-of-function mutants of the thylakoid rhodanase-like protein TROL (*trol*), pre-acclimated to either dark, growth-light, or high-light conditions, were used to probe the strength of FNR-membrane associations. Detergents β-DM (decyl-β-D-maltopyranoside) or β-DDM (n-dodecyl-β-D-maltopyranoside) were used to test the stability of FNR binding to the thylakoid membranes, and to assess different membrane domains containing FNR. Further, the extraction conditions mimicked pH status of chloroplast stroma during changing light regimes. Plants without TROL are incapable of the dynamic FNR recruitment to the photosynthetic membranes.

**Specifications Table**

**Table:** 

Subject area	*Biology, Biochemistry*
More specific subject area	*Protein interactions*
Type of data	*Western blots*
How data was acquired	*SDS-PAGE, Western transfer, ECL*
Data format	*Raw and analysed*
Experimental factors	*Arabidopsis thaliana (L.) ecotype Columbia (Col-0, WT) plants and At4g01050 knock-out mutant line, trol, were grown either in dark or under 80 µmol photons m*^*−2*^*s*^*−1*^*(GL) or 250 µmol photons m*^*−2*^*s*^*−1*^*(HL), respectively. Intact Arabidopsis chloroplasts were isolated from 3–4 week old plants and analysed by using SDS-PAGE for TROL-FNR complex stability.*
Experimental features	*For TROL-FNR complex dynamics investigation, thylakoids were isolated from intact Arabidopsis chloroplasts, separated by ultracentrifugation into insoluble and soluble fractions, treated by nonionic detergents at different pH, analysed by SDS-PAGE, Western transfer, immunodecorated with α-FNR antibody, and finally detected by semiquantitative ECL.*
Data source location	*Zagreb, Croatia*
Data accessibility	*Data is supplied in this article*

**Value of the data**

• Data assess alternative membrane binding and release of chloroplast FNR, or its association with different membrane complexes.
• Recruitment of FNR to thylakoids was tested on WT or *trol* Arabidopsis plants pre-acclimated to different light conditions.
• Biological function of FNR-membrane association in the context of photosynthetic electron flow regulation was addressed.

## 1 Data

**Fig. 1:**
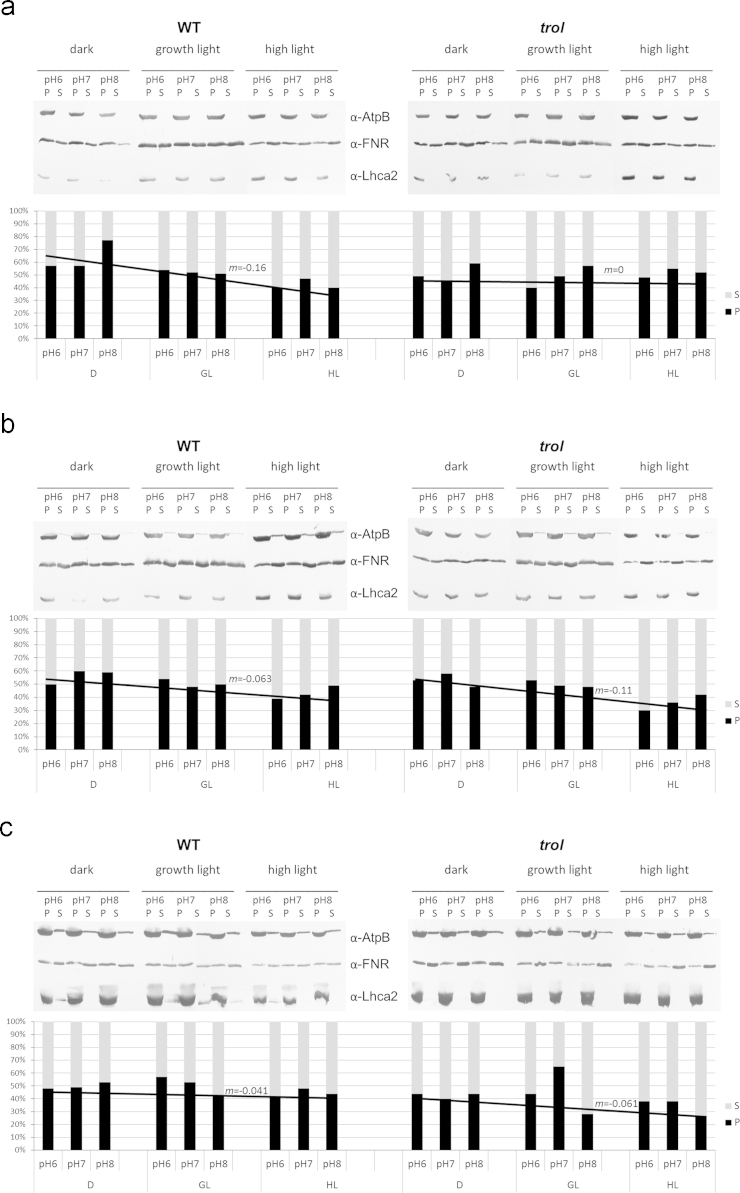
The distribution of FNR in the thylakoids of *Arabidopsis* WT and the *trol* plants grown under different light regimes and isolated under different pH conditions. Thylakoids isolated from *Arabidopsis* WT and *trol* plants were treated by different pH, in the absence of the detergent (a), or in the presence of β-DM (b), or β-DDM (c). Subsequently, membrane and soluble fractions were separated by ultracentrifugation into pellet (P) and supernatant (S), analyzed by using SDS-PAGE, followed by Western transfer and immunodecoration with the α-FNR. The α-AtpB and the α-Lhca2 were used as loading and extraction controls. ECL was used for final detection. Graphs represent the percentages of insoluble (P, black columns) and soluble fractions (S, gray columns), after the treatments. Trend lines were drawn according to the mean values of P/S for each data set to emphasize the differences in FNR distribution. The slope of the line (m) was calculated and indicated for each trend line. Graphs depict the representative data of the three independent experiments.

The data indicate that the absence of TROL protein influences the dynamic membrane association properties of chloroplast FNR. Thylakoids were isolated from plants acclimated to different light regimes by using buffers of different pH and containing either nonionic detergents decyl-β-D-maltopyranoside (β-DM) (Fig. 1b), or n-dodecyl-β-D-maltopyranoside (β-DDM) (Fig. 1c), or no detergent (Fig. 1a). The inclusion of β-DM or β-DDM was used to probe the stability of FNR binding to thylakoid supramolecular complexes and to assess different membrane domains containing FNR. The dynamism of TROL-FNR interaction was evaluated by quantifying FNR distribution between the membrane and the soluble fractions.

## 2 Experimental design, materials and methods

### 2.1 Plant material and growth conditions

*Arabidopsis thaliana* (L.) ecotype Columbia (Col-0) plants and *At4g01050* knock-out mutant line, *trol* [1], were grown on potting substrate (Stender, Germany) in the growth chamber (Kambič, Slovenia). Conditions were: 20° C, 80 µmol photons m^−2^ s^−1^ (Osram Flora, Osram, Germany) with a 16-h light photoperiod and a relative air humidity of 60% (day), or 70% humidity (night). Prior to analyses, plants were pre-acclimated for two days either to dark, or to 80 µmol photons m^−2^ s^−1^ (GL), or to 250 µmol photons m^−2^ s^−1^ (HL) (Envirolite fluorescent lamps, Envirolite, Great Britain). The *trol* line has been characterized in detail previously [1].

### 2.2 Chloroplast isolation

Intact *Arabidopsis* chloroplasts were isolated from 3- to 4- week old plants as described by Vojta et al. [2]. Final sedimentation was achieved by centrifugation at 1,000*g* for 5 min. Sediment was resuspended in the buffer containing 330 mM Sorbitol and 20 mM Tris/HCl pH 8.4. Chloroplast concentration was set to 1 mg chlorophyll per 1 ml buffer. Chloroplast intactness and functionality was assessed by oxygen evolution measurements [3, data not shown] [3].

### 2.3 Thylakoid isolation and pH treatment

Isolated intact *Arabidopsis* WT and *trol* chloroplasts were subsequently lysed by incubating in 10 mM Hepes/KOH pH 7.6 and 5 mM MgCl_2_, for 30 min on ice. Thylakoids were separated from stroma by centrifugation for 20 min at 50,000*g*, at 4 °C, and treated by using different pHs. Thylakoids corresponding to 20 µg chlorophyll were re-buffered in 100 mM sodium phosphate buffer (pH 6.0, 7.0 or 8.0), 10 mM MgCl_2_, 1 mM EDTA, in the absence of the detergents, or in the presence of 0.1% β-DM (v/v) or 0.1% β-DDM (v/v), and incubated for 10 min on ice, followed by additional 30 min at 25 °C, as described [4]. Subsequently, samples were separated by ultracentrifugation for 20 min at 50,000*g* at 4 °C into insoluble and soluble fractions, resolved by SDS-PAGE, followed by Western transfer and immunodecorated with the FNR antibody (as well as the α-AtpB and the α-Lhca2, as loading and extraction controls). Final detection was carried out by enhanced chemiluminescence (ECL) (Fig. 1). Negatives were scanned with the Hewlett-Packard flatbed scanner and the band intensities were quantified by using ImageJ program (NIH, USA).
