# Supplementary material for: Data supporting the absence of FNR dynamic photosynthetic membrane recruitment in trol mutants
Source: Data Brief. 2016 Feb 26;7:393–6. doi: 10.1016/j.dib.2016.02.044 (PMC4781999; doi:10.1016/j.dib.2016.02.044)
Supplement: Supplementary file 1 — Supplementary material [file mmc1.docx]

**Conflict of interest declaration**

Conflicts of interest: none.
